# Gene therapy and mRNA drugs approach for mitochondrial OXPHOS deficiencies

**DOI:** 10.1016/j.ymthe.2025.09.036

**Published:** 2025-09-23

**Authors:** Caterina Garone, Silvia Sabeni, Sara Carli

**Affiliations:** 1 Department of Medical and Surgical Sciences, Alma Mater Studiorum University of Bologna, 40138 Bologna, Italy; 2 UOC Neuropsichiatria dell’età Pediatrica, IRCCS Istituto delle Scienze Neurologiche di Bologna, 40138 Bologna, Italy

**Keywords:** mitochondria, mitochondrial disease, gene therapy, complexes, ATP, RNA drugs, AAV

## Abstract

Mitochondrial disorders are a clinically heterogeneous group of diseases due to defects in nuclear or mitochondrial DNA-encoded genes leading to mitochondrial dysfunction and oxidative phosphorylation deficiency in the affected tissues. The dual genetic controls, the biochemical heterogeneity, and the clinical variability challenge the development of effective treatment. In this review, we focus on gene therapy and mRNA drug approaches for nuclear-encoded gene defects causing isolated, combined, or multiple oxidative phosphorylation defects and mitochondrial-encoded gene defects for which a gene replacement approach has been tested, and on the allotopic expression of mtDNA genes. An overview of the available *in vitro* and *in vivo* disease models and pre-clinical data of safety and efficacy is provided and highlights challenges in correcting the biochemical defect in the most affected tissues. Future perspectives with the use of novel gene editing approaches or gene replacement delivery with nanoparticles are also considered as a novel strategy for treating mitochondrial disorders.

## Graphical abstract

**Figure:**
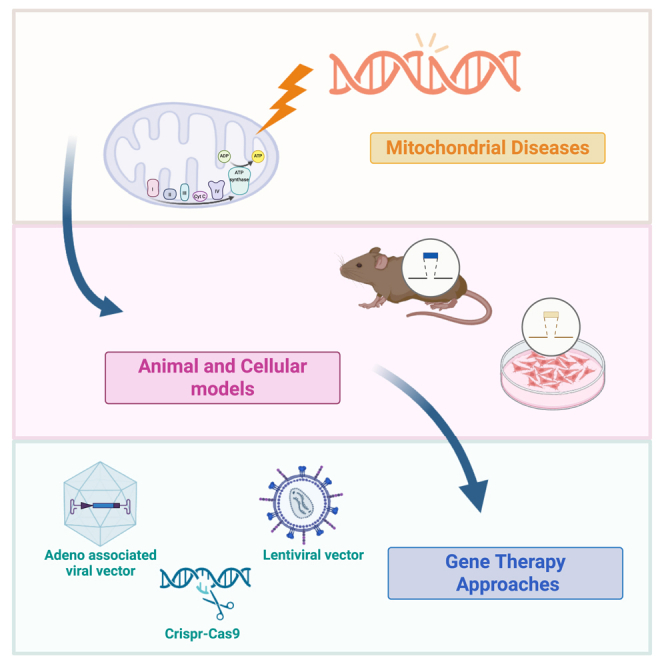

## Teaser

Garone and colleagues explore the potential of gene therapy and mRNA drugs in treating mitochondrial disorders, reviewing *in vivo* and *in vitro* models. They emphasize the challenges that remain to be addressed in improving the therapeutic approach, which represents a promise for these complex and currently incurable diseases.

## Introduction

**Figure 1:**
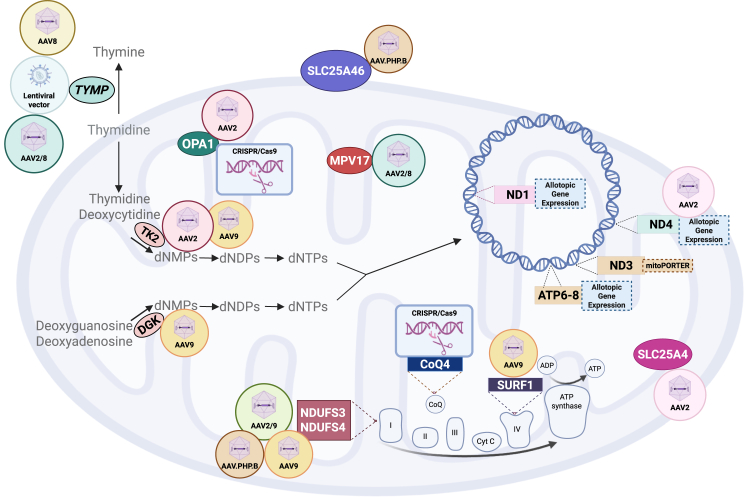
Graphical representation of gene therapies applied to mitochondrial disorders due to nuclear and mitochondrial DNA mutations AAV, adeno-associated viral vector; dNMPs, deoxynucleotides monophosphate; dNDPs, deoxynucleotides diphosphates; dNTPs, deoxynucleotide triphosphates.

Mitochondria are dynamic double-membrane organelles whose main function is ATP production through oxidative phosphorylation (OXPHOS), although they have multiple additional roles in cellular activity.^1^ Mitochondria have their genome (mitochondrial DNA, mtDNA) of approximately 16.5 kb, containing 37 genes encoding for 13 mitochondrial complex subunits, 22 tRNAs, and 2 rRNAs.^2^ In addition to mtDNA-encoded genes, ∼1,200 proteins required for the remaining OXPHOS subunits, mitochondrial homeostasis, and function are encoded by the nuclear genome (nDNA).^3^^,^^4^ Pathogenic variants of nDNA or mtDNA-encoded genes cause mitochondrial disorders, a group of genetic diseases that are individually rare, but with an overall prevalence of about 1 in 4,300 in adults.^5^ Genetically, mtDNA variants are subject to maternal inheritance, presence in heteroplasmy or homoplasmy in different tissues, and a threshold effect for biochemical and clinical impact; defects of nuclear genes encoding mitochondrial proteins are instead Mendelian inherited. Clinically, they present a variety of signs and symptoms potentially affecting any tissues, at any age. Biochemically, they are characterized by OXPHOS deficiency, which can be isolated to only one mitochondrial respiratory chain complex (e.g., complex I (CI)) or spread to multiple mitochondrial respiratory chain complexes in the affected tissues.^6^^,^^7^ The lack of a genotype-phenotype correlation further complicates the clinical picture and challenges diagnosis, management, and the development of experimental therapies. Gene therapies or RNA therapeutics targeted to a specific gene defect or a common pathway have been designed, and their clinical efficacy and safety have been tested in *in vivo* and *in vitro* models for mitochondrial disorders. However, the theoretical value of that treatment is yet to be optimal due to challenges in delivering the treatment to the brain, limited efficiency and efficacy in restoring the bioenergetic defect and clinical phenotypes, and potential toxicity of the specific system delivery. In this review, we present an overview of *in vivo* and *in vitro* models where these therapies have been tested, highlighting the major progress made in overcoming challenges (Table 2; Figure 1, graphical representation). Given the different approaches required for targeting the mtDNA, we focus this review on mitochondrial disorders due to nuclear gene defects and the allotopic expression of the mtDNA gene, whereas strategies aiming to shifting heteroplasmy or editing the mtDNA are not discussed. We explore the most promising strategies that have been developed, discussing their mechanisms of action and potential for clinical application with the final aim of providing a comprehensive understanding of the potential of gene therapy in treating mitochondrial diseases.

## Isolated CI deficiency

The first complex responsible for the generation of 40% of the proton-motive force is the NADH ubiquinone oxidoreductase or CI, which reduces coenzyme Q_10_ by transferring electrons from NADH and concurrently translocating protons in the intermembrane space.^36^ CI is composed of 14 core subunits, 7 encoded by the nuclear DNA (*NDUFV1*, *NDUFV2*, *NDUFS1*, *NDUFS2*, *NDUFS3*, *NDUFS7*, and *NDUFS7*) and 7 encoded by the mitochondrial DNA (*ND1*, *ND2*, *ND3*, *ND4*, *ND4L*, *ND5*, and *ND6*). They are organized in an L-shape, divided into three modules: modules Q and N forming the arm and facing the matrix, which is responsible for the transit of electrons to the coenzyme Q_10_; the intermembrane module P assists the passage of protons into the intermembrane space.^37^ Thirty-one accessory subunits of CI have been identified so far, but the precise function of each one remains to be investigated. Studies on modified HEK293T to research the consequences of a single knockout of each subunit led to the identification of 25 subunits required in the assembly and one subunit indispensable for cell viability.^38^ Pathogenic variants in genes encoding CI subunits or assembly factors can cause a variety of clinical phenotypes, from encephalopathy to myopathy to multisystemic diseases.^39^ The most frequent clinical syndromes are represented by Leigh syndrome (Mendelian inherited MIM: 256000 or mitochondrial inherited MIM: 500017), Leber hereditary optic neuropathy (LHON, MIM: 535000), and mitochondrial encephalopathy and lactic acidosis stroke-like disease (MELAS, MIM: 540000).

Adenovirus-associated gene therapy approaches have been developed in knockout (KO) mouse models for the nuclear genes *Ndufs4*^32^^,^^40^ and *Ndufs3*,^12^ both reproducing the human disease with a Leigh syndrome phenotype. Details on the generation and characterization of the mouse models are summarized in Table 1.

**Table 1:** Mouse models used for gene therapy approaches

Gene	Encoded protein	Disease	Disease model	Generation of the model	Phenotype	References
*NDUFS4*	NADH: ubiquinone oxidoreductase subunit S4	Leigh syndrome Mendelian inherited, MIM: 256000	*Ndufs4*^−/−^ KO mice	Cre/loxP-mediated homologous recombination	• onset up to P21 with body weight growth delay and hair loss as initial signs of the disease • rapid disease progression by PD30 • reduction of the fully assembled CI in the heart, muscle, brain, kidney, liver, pancreas, and lung • behavioral abnormalities (no socializing, grooming, and feeding), ataxia, seizures, lethargy • hypothermia (∼2°C less than control) • survival: 50 days	Van De Wal et al.^36^; De Haas et al.^40^
*NDUFS3*	NADH: ubiquinone oxidoreductase subunit S3	Leigh syndrome Mendelian inherited, MIM: 256000	*Ndufs3*^−/−^ skeletal muscle-specific KO	Cre/loxP-mediated homologous recombination	• reduced body weight • motor abnormalities • survival: 8 months • mitochondrial myopathy • increased SDHA activity • reduced CI activity in muscle • abnormal mitochondrial morphology • survival: 9–14 weeks	Pereira et al.^12^
*ND4*	CI subunit	LHON, MIM: 535000	R304H mouse	site-directed mutagenesis	• swelling of the optic nerve • altered mitochondrial morphology • progressive retinal disruption	Qi et al.^41^
*SURF1*	CIV assembly genes	mitochondrial CIV deficiency, nuclear type 1, MIM: 220110 Leigh syndrome Mendelian inherited, MIM: 256000	*Surf1*^−/−^ KO mouse	Cre/loxP-mediated homologous recombination	• mild phenotype • motor impairment • COX deficiency and increased SDH was in skeletal muscle • reduction of CIV activity in the liver, brain, muscle, and fibroblasts; high blood lactate • the clinical phenotype was very modest, with a slight reduction of performance of KO in the rotarod test compared with WT • survival: 793 days	Dell’Agnello et al.^42^
*OPA1*	mitochondrial GTPase	autosomal dominant optic atrophy, MIM: 165500	*Opa1*^delTTAG^	Cre/loxP-mediated homologous recombination	• ataxia and motor impairment • deafness • neuronal degeneration • decreased OPA1 levels in the brain, retina, optic nerve, and heart • diminished rIV activity in the retina, optic nerve, and glycolytic muscle • increased number of mitochondria, vacuolated shapes, and no cristae organization	Sarzi et al.^20^
*SLC25A46*	solute carrier family 25 member 46	Leigh syndrome Mendelian inherited, MIM: 256000	*Slc25a46*^−/−^ KO mouse	CRISPR-Cas9 technology (c.992-1037del; p.Leu331fs∗346)	• onset: P14 with gait imbalance and reduced body growth • loss of ambulation, muscle atrophy, peripheral neuropathy, and seizures • small brain at the MRI • loss of dendrites and whole Purkinje cells • larger mitochondria with disorganized cristae • retinal ganglion cell loss • survival: 2–8 months	Li et al.^43^
*SLC25A4*	adenine nucleotide translocator 1	cardiomyopathy, MIM: 617184, MIM: 615418	*Slc25a4*^−/−^ KO mouse	Cre/loxP-mediated homologous recombination	• ragged-red fibers in skeletal muscles • cardiac hypertrophy • reduction in CIII respiration rate • motor impairment	Graham et al.^44^
*TYMP*	thymidine phosphorylase	MNGIE, MIM: 603041	*Tymp*^−/−^/*Upp1*^−/−^ dKO mouse	Cre/loxP-mediated homologous recombination	• mild phenotype • elevated Thd and dUrd levels in spleen, kidney, lung, and muscle and to a lesser extent in liver, small intestine, heart, and brain • mtDNA depletion in the brain • reduced CI and IV activity	López et al.^45^
*TYMP*	thymidine phosphorylase	MNGIE, MIM: 603041	Thd/dUrd-exposed *Tymp*^−/−^/*Upp1*^−/−^ dKO mouse	Cre/loxP-mediated homologous recombination plus administration of thymidine (68.6 mM) and deoxyuridine (72.3 mM) at a standard concentration of 16.6 mg/mL in drinking water up to 2 years	• reduced body weight • motor impairment • decreased mtDNA in the brain and small intestine • mitochondrial dNTP pool unbalance • Thd and dUrd tissue concentrations >100-fold over the controls • leukoencephalopathy	Garcia-Diaz et al.^46^
*MPV17*	Mpv17	hepatocerebral MDS, MIM: 256810	*Mpv17*^−/−^ KO mouse	retroviral construct to silence the gene	• decreased mtDNA in the liver and muscle • reduction in complexes I–IV • abnormal mitochondrial cristae morphology in hepatocytes from P60 • increased AST, ALT, and CK enzymes • subclinical mitochondrial myopathy from 1 year old • survival: 635 days	Viscomi et al.^47^
*DGUOK*	deoxyguanosine kinase	hepatocerebral MDS, MIM: 601465	*Dguok*^−/−^ KO mouse	Cre/loxP-mediated homologous recombination	• impaired body growth • motor impairment • early-onset mtDNA depletion in the liver, skeletal muscle, spleen, and brain • multiple OXPHOS deficiencies in liver, isolated CIV deficiency in brain • elevated citrate synthase activity • increased of blood ALT, AST, ALP, and amino acids (threonine, glycine, arginine, and methionine) • survival: 42 weeks	Zhou et al.^48^
*TK2*	thymidine kinase 2	MDS myopathy, Tk2-related**,** MIM: 609560	*TK2* H126N KI mouse	Cre/loxP-mediated homologous recombination	• onset: P10 with tremor, gait unbalance and reduced body growth • decreased TK2 activity in brain, liver, heart, and muscle • mtDNA depletion in brain, spinal cord, heart, muscle, and kidneys • survival: 39 days	Akman et al.^49^; Lopez-Gomez et al.^34^

ALP, alkaline phosphatase; ALT, alanine aminotransferase; AST, aspartate transaminase; dKO, double knockout; DOA, dominant optic atrophy; KI, knockin; KO knockout; LHON, Leber hereditary optic neuropathy; MDS, mitochondrial depletion syndrome; MNGIE, mitochondrial neurogastrointestinal encephalopathy; MRI, magnetic resonance imaging; mtDNA, mitochondrial DNA; OXPHOS, oxidative phosphorylation; WT, wild-type.

Adeno-associated viral vector (AAV) gene therapy was first attempted with the AAV2/9 serotype, delivering the human wild-type *NDUFS4* cDNA under the promoter of the cytomegalovirus (CMV) and combining different routes of administration in the *Ndufs4 KO* mouse.^51^ Single retro-orbital intravenous (i.v.) injection at 2 × 10^12^ viral genomes/mouse (vg/mouse) at PD21 or i.v. temporal vein injection at 1 × 10^12^ or 2 × 10^12^ vg/mouse in newborn mice did not show efficacy at the phenotypical and molecular genetics level. At the biochemical level, a slight increase in CI and citrate synthase activities was demonstrated in skeletal muscle tissue with the 2 × 10^12^ vg/mouse i.v. temporal vein injection. Intracerebroventricular (i.c.v.) injection at both 1.5 × 10^11^ and 3 × 10^11^ vg/mouse was able to obtain a good transduction level in the brain with a dose-related effect: CI activity was 8% in untreated mice, whereas, respectively, 42% and 65% at the low and high doses in the brain. The best results at phenotypical, molecular genetics, and biochemical levels were obtained through a double injection with retro-orbital i.v. administration at 2 × 10^12^ vg/mouse and i.c.v. at 3 × 10^11^ vg/mouse in newborns. Treatment ameliorated the phenotype with increased body weight, improved motor coordination, and longer survival. At the molecular level, the treatment was not able to completely restore CI protein level and activity: Ndufs4 protein level was present in the heart, muscles, and brain but not in the liver; CI activity was restored to wild-type levels in muscle and heart, and up to 70% in the brain compared with controls.^8^

A second gene therapy approach has been tested with the AAV.PHP.B serotype, deemed to have higher efficiency in crossing the blood-brain barrier. Its strong neurotrophic property is due to the addition of a motif of seven amino acids, TLAVPFK, in the hypervariable region VIII of AAV9. This region has the power to bind to a glycosylphosphatidylinositol-anchored protein, resident on brain endothelial cells, known as lymphocyte antigen 6 complex locus A (LY6A).^52^ Mice were treated between PD26 and PD28 through a single-tail i.v. injection with 10^12^ or 2 × 10^12^ vg/mouse. No difference was observed between the two dosages. Ten days after the injection it was observed a normalization of the body weight and an improved performance on the rotarod test compared with untreated mice. Moreover, treated animals showed a prolonged median lifespan (100 days) and a survival rate of up to 1 year in 30% of the cases. Recovery of the fully assembled CI was reported in brain homogenates, together with CI activity. The authors also attempted an i.v. injection of 10^12^ vg/mouse in P1 mice, but they noticed poor brain expression of hNDUFS4 and a survival rate comparable with that of untreated mice, possibly due to the lower expression of LY6 in newborns compared with young adult mice.^9^

Reynaud-Dulaurier et al.^10^ obtained comparable results by administering 10^12^ GC in the retro-orbital sinus at 1 month of age. The most important phenotypical result was an increase in the lifespan that reached 250 days for at least 50% of the treated mice. Moreover, the authors analyzed for the first time the percentage of the mice showing epilepsy and found that only 5% of the KO injected with AAV-*Ndufs4* manifested seizures compared with 23% of the untreated KO.^10^ The safety of the AAV.PHP.B vector was further investigated in non-human primates (NHPs) (adult rhesus macaques) with the i.v. administration of both 2 × 10^13^ and 7.5 × 10^13^ GC/kg (genome copies/kg) GFP-AAV.PHP.B. Although the lower dose did not cause complications, it was reported that transduction efficacy in the central nervous system (CNS) was limited compared with that previously observed in C57BL/6J mice. To explain this discrepancy, the authors identified a polymorphism of LY6A, which is present selectively in the C57BL/6J mouse strain but not in BALB/cJ mice, NHPs, or humans. On the other side, the higher dose led to severe side effects such as elevated transaminases, thrombocytopenia, and bleeding, and a low transduction efficacy was reported. Altogether, these findings limited the human translation of *NDUFS4*-AAV.PHP.B therapy.^53^

A recombinant self-complementary double-stranded AAV9-*NDUFS4* (scAAV9^NDUFS4^) was exploited by Corrà et al. A double injection intravascular and i.c.v. of 10^11^ viral particles was performed in PD1 mice. A single mouse died at 85 days, while the remaining four mice lived for 9 months. In brain homogenates, comparable levels of NDUFS4 were observed between WT and KO-treated animals.^11^ The striking results obtained in four out of five treated mice support the potential efficacy of this treatment and open the way for further study in other animal models before human translation. On the other hand, the severe neurological phenotype of only one mouse that died also suggests that genetic background or prenatal disease stage might influence the outcome of gene therapy.

RNA drugs have also been hypothesized as a treatment for CI deficiency to increase mitochondrial biogenesis and clearance of damaged mitochondria.^54^ Indrieri et al. attempted to rescue the phenotype in *Ndufs4*^−/−^ mouse modulating microRNA (miRNA) expression of miR-181a and miR-181b, both present in CNS and retina, and able to bind and downregulate different targets. Specifically, they can modulate PPARGC1A and NRF1, which have a role in biogenesis, COX11 and COQ10B, involved in the respiratory chain assembly, and PARK2, which controls mitophagy. To investigate the effect of downregulation of miR-181a/b, the authors crossed *Ndufs4*^−/−^ with mice harboring a deletion in the clusters coding these two miRNAs, thus obtaining *Ndufs4*^−/−^*/miR-181a/b-1*^−/−^ mice. They presented an increased number of retinal ganglion cells (RGCs) with an enhancement of visual acuity. From a molecular point of view, the *Ndufs4*^−/−^*/miR-181a/b-1*^−/−^ mice showed higher levels of *Nrf1* and *Park2* mRNAs compared with *Ndufs4*^−/−^ animals. Electron microscopy displayed an augmented number of mitochondria, and cristae of RGCs appeared more defined and structured, while biochemical analysis revealed an increase in CI, CII, and CIV activities. These results demonstrated that the synergic effect on biogenesis and clearance exerted by the downregulation of miR-181a/b has a protective role in mitochondrial disorders.^55^^,^^56^ To deliver miR-181a/b as a treatment strategy, a miRNA sponge has been designed and used in a model of inherited retinal diseases in which mitochondrial dysfunction is reported as a primary disease mechanism.^57^ miRNA sponges are RNA molecules with tandemly miRNA binding sites, able to sequester miRNAs from their endogenous target resulting in long-term silencing.^58^ Results from this study demonstrated the safety and efficacy of targeting mitochondrial dysfunction in a tissue-specific manner and paved the way for the application of the sponge approach to silence miR-181a/b also in the *Ndufs4*^−/−^ mouse model.

AAV gene therapy with the AAV9 serotype has also been tested in *Ndufs3*^−/−^ skeletal muscle-specific KO mice (smKO) (Table 1).^12^ To revert the myopathic phenotype, the rAAV9- *Ndufs3* was administered with a single retro-orbital injection at 1.25 × 10^15^ vg/kg concentration in PD15-18 mice. Motor functions were reestablished 1.5 months after the injection, no signs of myopathy were reported, and the survival was extended up to 15 months. The treatment restored the Ndufs3 protein in the skeletal muscle of *Ndufs3* smKO mice and normalized histochemical (SDH) and histological muscle abnormalities. Similar results were obtained by applying the same procedure to symptomatic mice (P60). The results highlighted that rAAV9-*Ndufs3* administration was effective in preventing the onset of myopathy in the smKO mouse model.^12^

Altogether, these findings demonstrate that AAV gene therapy has limited efficacy in the brain when administered by i.v. injection compared with i.c.v. delivery, even when using the AAV2/9 serotype that has specific tropism for CNS in mitochondrial disease models with encephalopathy, such as the *Ndufs4*^−/−^ mouse. A dose-related effect with i.c.v. injection is also suggested, with no reported toxicity.^8^ The reported studies demonstrated that the AAV.PHP.B vector, although effective in delivering the wild-type gene, has no translational value in humans,^53^ whereas encouraging results have been observed with scAAV9, but further studies are needed to confirm the long-term efficacy.^11^ Notably, early intervention using rAAV9 appears to be more effective than AAV2/9, even if treatment of symptomatic mice has been shown to reverse the clinical phenotype, at least in the smKO *Ndufs3* mouse model. Nonetheless, it will be important to evaluate this therapeutic approach in additional full KO models to assess its efficacy in the CNS.^12^

The gene therapy approach for mtDNA genes faces additional challenges due to the delivery to the organelle and the variable level of mtDNA heteroplasmy. To overcome these issues, allotopic expression has been considered for the transfer and expression of mtDNA genes in the nucleus. The design requires a codon optimization considering the diverse and singular genetic code of mtDNA versus the universal genetic code and the presence of a mitochondrial target sequence for the localization of the protein into mitochondria after translation. To correct the defect, the system should also allow the assembly of the wild-type protein subunit into the mitochondrial complex. Although the attempt has been performed for all 13 polypeptides encoded by mtDNA, only for a few of them was it possible to allotopically re-express the wild-type gene and correct the defect in *in vitro* and *in vivo* disease models. Limitations to this genetic approach can be due to the hydrophobicity and the competitive interaction with the defective protein in the complex assembly.^59^

For CI deficiency due to mtDNA variants, allotopic expression of ND1 or ND4 genes was experimented with preliminarily *in vitro* models, showing the ability to express the wild-type protein and rescue the complex activity.^15^ The allotopic gene therapy approach was further exploited for the *ND4* genes by experimenting with AAV delivery in *in vitro* and *in vivo* models.^15^

To use AAV and address the delivery to mitochondria, Yu et al. developed AAV2 with a mitochondrial targeting sequence (MTS-AAV2) and wild-type cDNA of *ND4.* Preliminary results were obtained *in vitro* in cybrids harboring the common variant G11778A. Two days after the transfection, cybrid cells were selected in a glucose-free galactose medium for 5 days to eliminate those that did not receive the vector. Fluorescence microscopy confirmed the localization of AAV into mitochondria. ATP synthesis in treated cells presented an increase of 48%. To ameliorate the efficiency, an AAV with a self-complementary AAV (scAAV) inserted with both forward and reverse strands of *ND4* DNA was used, and the results in ATP synthesis increased 4-fold compared with the previous AAV2 treatment.^13^ *In vivo*, studies were carried out on an available mouse model carrying the R340H variant on the *Nd4* gene.^41^ Mice were injected with viral vectors in the right eye into the vitreous body. Three and 9 days after injection, MTS-AAV2 was able to translocate ND4 into mitochondria. Optic atrophy was ameliorated with a significant difference in optic nerve diameter between the two eyes. In conclusion, the authors indicated the MTS-AAV2 as a translatable gene therapy for ND4 mutation causing LHON.^13^

A few years later, Koilkonda et al. tested the MTS-scAAV2 in the *Nd4* G11778A mouse model to assess its efficacy and safety. The authors administered 1 μL of scAAV2-*ND4* (5.02 × 10^12^ particles/mL) into both the right and left eye. Pattern electroretinogram amplitude was rescued at 2 and 12 months post-injection compared with untreated KO mice. Histological analysis of the retro-orbital area showed that the treatment was successful in preventing optic atrophy. Additional studies on NHPs showed no inflammatory response or immunogenicity in all major organs^14^

Yamada et al. designed a therapeutic RNA containing an optimized copy of the mRNA of *ND3* and delivered it into the patients' cell line with T10158C through a liposome called MITO-Porter, able to exploit the membrane fusion. Untreated cells had a mutational load of around 80%, while, after treatment, there was a reduction, with a residual heteroplasmy between 10% and 20% in 48 h. Consequently, maximal mitochondrial respiratory activity was significantly increased.^60^

## Isolated CII and CIII

CII, also known as succinate ubiquinone oxidoreductase, is the smallest complex of the respiratory chain and the sole one to be entirely encoded by the nuclear DNA. It is a tetramer (SDHA, SDHB, SDHC, SDHD) involved in the oxidation of succinate into fumarate, reducing FAD to FADH_2_. Most common pathogenic phenotypes derive from mutations in the *SDHA* gene, leading to Leigh syndrome. Mutations in the other subunits or some assembly factors contribute to less frequent diseases generally characterized by hypotonia and/or ataxia.^61^ CIII, also named ubiquinol cytochrome *c* oxidoreductase, transports electrons from ubiquinol to cytochrome c, while protons arrive at the intermembrane space. The structure is composed of 11 subunits, of which only one is encoded by the mtDNA and the other by the nuclear DNA. Isolated CIII deficiencies are rare and present a wide range of symptoms typical of mitochondrial diseases, such as liver failure or hyperlactatemia.^62^ Although in the literature, some therapeutic approaches have been described, none of them include gene therapy so far.

## Isolated CIV deficiency

CIV, or cytochrome *c* oxidase, is responsible for the oxidation of the ferrocytochrome C in favor of the reduction of oxygen.^63^ Fourteen subunits compose this complex: 11 of them are nuclear-encoded, and 3 of them have a mitochondrial origin. Several assembly factors have been recognized, such as SURF1, SCO1, SCO2, COX10, and COX15.^64^ Mutational variants in nuclear or mitochondrial gene-encoding CIV subunits lead to the onset of several disease phenotypes such as MELAS syndrome, myopathy, or Leigh syndrome. In particular, although genetically heterogeneous, Leigh syndrome could be due to mutations in the CIV assembly genes *SURF1*, causing a disorder characterized by progressive encephalopathy, ataxia, and psychomotor regression (MIM: 220110).^65^

The scAAV9 gene therapy approach has been tested in the *Surf1* KO mouse model (Table 1).^42^ The authors elicited the ubiquitous hybrid chicken β-actin promoter and the bovine growth hormone polyadenylation promoter, CBh, to ensure a massive and widespread expression of the transgene. The positive effect of the treatment was found to be independent of the administration doses (8 × 10^11^ and 2 × 10^11^ vg) and routes (intrathecal or intrathecal plus i.v.) in 4-week-old mice. *Surf1* mRNA was expressed in different CNS regions, such as the striatum, hypothalamus, thalamus, midbrain, cerebellum, and lumbar spinal cord, but also regions distant from the injection site, such as the cervical spinal cord. Additionally, lactate was significantly reduced in treated mice. No side effects were recorded up to 1 year of treatment, even for the high dose of 8 × 11^11^ vg/mouse, thus implying the safety of both dosages.^16^

## Isolated CV deficiency

CV, also called ATP synthase, is composed of 18 protein subunits (16 nDNA encoded and 2 mtDNA encoded) and its main role is the conversion of ADP into ATP by a proton gradient generated by CI to CIV.^66^ Pathogenic variants in nuclear subunits lead to developmental and epileptic encephalopathy (MIM: 619970). In mtDNA, the *ATP6* 8993T>G and 156T>C are the most frequent variants, leading to neuropathy, ataxia, and retinitis pigmentosa (MIM: 551500) and maternal inherited Leigh syndrome (MIM: 500017).^67^ Although *in vitro* and *in vivo* studies showed the ability of allotopic expression of both mt*-ATP6* and mt*-ATP8* genes in rescue CV deficiency,^67^ no AAV-based therapy has been developed for CV-related disease so far.

## Other approaches

Alternatively to allotopic gene expression, strategies for shifting the heteroplasmic level of mtDNA variants have been developed. The aim was to specifically target mtDNA molecules carrying a pathogenic variant, recognize the mutated site, induce double-strand breaks (DBs), and, in the absence of an efficient mechanism of repair, cleave the defective molecule. This allows the replication of only wild-type molecules of mtDNA and consequently the replacement of damaged mtDNA. Mitochondrial restriction endonuclease (mitoRe) was first used to generate site-specific DNA DBs to cleave mtDNA sequences, rapidly eliminate mtDNA, and promote heteroplasmy shifting. However, the technology was able to target only a few pathogenic mtDNA mutations with a specific haplotype, which resulted in unique restriction endonuclease recognition sites. To overcome these limits and target any variants, different strategies have been designed and experimented with *in vitro* and *in vivo* models (mt-zinc finger nuclease, mitoTALENS, mitoTev-TALE) with promising results in terms of safety and efficacy. Additional challenges regarding the packaging into AAV and delivery have been additionally exploited by using technology such as mitoARCUS, organized in a monomer or the meganucleases, relatively small enzymes with long DNA recognition sites (22 bp), offering a broad potential for sequence specificity.^68^^,^^69^ Recently, innovative approaches of precise base editing using DddA-derived cytosine base editors or a mitochondrial base editor (mtBE) combining a mitochondria-targeted programmable TALE-binding protein with nickase MutH or Nt.BspD6I(C), and either the single-stranded DNA-specific adenine deaminase TadA8e or the cytosine deaminase APOBEC1 and uracil glycosylase inhibitor have been exploited.^70^^,^^71^ These tools are able to catalyze A to G or C to T conversion at specific mtDNA sites without requiring gRNA. The main advantages of base editors include high precision and irreversible base modification; however, they present issues such as potential off-target editing and base substitution limitation.^72^ Detailed description and critical analysis of *in vivo* and *in vitro* studies using these technologies are beyond the scope of this review since their approach is not based on gene expression modulation.

## Combined OXPHOS deficiencies

### Defects in mito-dynamics and translocators

Mitochondria are highly dynamic organelles, with a tight control of fusion and fission mechanisms to maintain homeostasis and the correct number, morphology, and length of the different populations of mitochondria in cells. Fusion permits the exchange of DNA and metabolites between close mitochondria, including damaged or senescent ones, promoting their survival, and it takes place on both inner and outer membranes (IMMs and OMMs). The external process is played by two GTPase family proteins, mitofusin 1 (MFN1) and mitofusin 2 (MFN2), with a role in the fusion of the two OMMs, while the optic atrophy protein (OPA1) promotes the fusion of the IMM.^73^ On the other hand, mitochondrial fission is driven by dynamin-related protein 1 (DRP1) and inverted formin 2 (INF2). INF2 drives actin polymerization at constriction sites between mitochondria and the ER membrane through actin polymerization. Then, DRP1 is translocated from the cytosol to the OMM and, with its GTPase activity, promotes mitochondrial fission.^74^ Defects in genes controlling the mitochondrial dynamics can cause a quantitative and/or qualitative defect in mtDNA, and biochemically can be associated with a deficiency in one or more mitochondrial complex activities. At the clinical level, they are associated with neuromuscular and CNS degeneration. Pathological mutations in *MFN2* and *DRP1* cause different types of Charcot-Marie-Tooth disease (type 2A MIM: 609260; type 2M MIM: 606482), defined by progressive degeneration of peripheral sensory and motor axons, while mutations in *INF2* lead to renal focal segmental glomerulosclerosis (MIM: 256020). Moreover, pathogenetic variants in *OPA1* lead to *OPA1*-derived dominant optic atrophy (MIM: 165500), the most common hereditary optic neuropathy characterized by the loss of RGCs and visual impairments.^75^^,^^76^

Sarzi et al. generated a mouse model carrying the most common *OPA1* mutation in patients (c.2708_2711delTTAG) (Table 1).^77^ AAV2/2 serotype was chosen for its specificity for RGCs and was charged with the human cDNA of *OPA1* gene isoform 1 under the control of the CMV promoter in this mouse model. Treatment was administered in 3-month-old mice by intravitreal injection at a concentration of 1.6 × 10^11^ vg/mL. Eight months after treatment, visual acuity reached a comparable level with controls, and RGBs counting was increased in treated mice.^20^

A CRISPR-Cas9 editing approach was also tested in an *in vitro* model. Sladen et al. generated induced pluripotent stem cells (iPSCs) from patient-derived fibroblasts presenting *OPA1* mutation in position c.1334G>A (p. Arg445His). Two different guide RNAs (gRNAs) were designed to target the variant very closely: gRNA_1_ 15 bp upstream and gRNA_2_ 14 bp downstream. Edited clones were selected by G homozygosity in c.1334. Confocal microscopy studies showed the restoration of the mitochondrial network while Seahorse analyses revealed correction in ATP production, confirming efficacy also in mitochondrial bioenergetics.^21^

Mitochondrial carriers such as SLC25A46 cooperate with fusion and fission processes.^78^^,^^79^ Mutations in *SLC25A46* have been reported as causative for different diseases, such as pontocerebellar hypoplasia type 1E (MIM: 619303), neuropathy, hereditary motor and sensory type VIB (MIM: 616505) and Mendelian inherited Leigh syndrome (MIM: 25600).^80^

To reach a CNS efficacy, AAV2-PHP.B containing the wild-type *Slc25a46* gene was administered to *Slc25a46*^−/−^ mice (Tables 1 and 2)^43^ via facial vein at PD2 in two different doses: 1 × 10^11^ and 2 × 10^11^ GC/g. A dose-dependent efficacy was demonstrated 51 days post-injection, with the higher dose showing a greater positive effect in prolonging the lifespan and increasing the body weight compared with untreated mice. At PD40, it was demonstrated the ability of AAV-*Slc25a46* to prevent cerebellar neuropathy, with a decreased number of degenerating neurons and increased density of Purkinje cells in the cerebellum; electron microscopy also showed the restoration of the altered mitochondrial morphology. Optic atrophy ameliorated when the RGC layer was analyzed with both hematoxylin and eosin staining and immunostaining with the RGC-specific marker homeobox protein 3A (Brn3a). Biochemically, treated mice showed a significant increase in CI and CIV in the heart and muscle tissue, together with a significant improvement in CII + CIII in the brain.^22^

**Table 2:** Gene therapy approaches on different models of mitochondrial disorders

Gene	Model	Gene therapy	Promoter	Administration route	Outcome	Efficacy	Time point of analysis	Safety	References
**Isolated CI deficiency**									

*NDUFS4*	*Ndufs4*^−/−^KO mouse	AAV2/9- *hNDUFS4*	CMV	2 × 10^12^ vg/mouse i.v. in P21	protein expression (WB)	↑ skeletal muscle, heart, liver	NA	no toxicity reported	Di Meo et al.^8^
					body growth	no	NA		
					motor coordination (Rotarod)	no	NA		
					survival	no improvement: 58 days	–		
*NDUFS4*	*Ndufs4*^−/−^KO mouse	AAV2/9- *hNDUFS4*	CMV	1 × 10^12^ vg/mouse i.v. in P1	protein expression (WB)	↑ skeletal muscle, heart	NA	no toxicity reported	Di Meo et al.^8^
					CI activity	↑ skeletal muscle and heart	NA		
					body growth	no	NA		
					motor coordination (Rotarod)	no	NA		
					survival	no improvement: 51 days	–		
*NDUFS4*	*Ndufs4*^−/−^KO mouse	AAV2/9- *hNDUFS4*	CMV	2 × 10^12^ vg/mouse i.v. in P1	protein expression (WB)	↑ skeletal muscle, heart	NA	no toxicity reported	Di Meo et al.^8^
					CI activity	↑ skeletal muscle and heart	NA		
					body growth	no	NA		
					motor coordination (Rotarod)	no	NA		
					survival	no improvement: 53 days	–		
*NDUFS4*	*Ndufs4*^−/−^KO mouse	AAV2/9- *hNDUFS4*	CMV	1.5 × 10^11^ vg/mouse i.c.v. in P1	protein expression (WB)	↑ brain	NA	no toxicity reported	Di Meo et al.^8^
					CI activity	↑ brain	NA		
					body growth	no	NA		
					motor coordination (Rotarod)	no	NA		
					survival	no improvement: 55 days	–		
*NDUFS4*	*Ndufs4*^−/−^KO mouse	AAV2/9- *hNDUFS4*	CMV	3 × 10^11^ vg/mouse i.c.v. in P1	protein expression (WB)	↑ brain	NA	no toxicity reported	Di Meo et al.^8^
					CI activity	↑ brain	NA		
					body growth	↑	NA		
					motor coordination (Rotarod)	↑	NA		
					survival	no improvement: 60 days	–		
*NDUFS4*	*Ndufs4*^−/−^KO mouse	AAV2/9- *hNDUFS4*	CMV	2 × 10^12^ vg/mouse i.v. coupled with 1.5 × 10^11^ vg/mouse i.c.v. in P1	protein expression (WB)	↑ brain, heart, muscle	NA	no toxicity reported	Di Meo et al.^8^
					CI activity	↑ heart, muscle, and brain	NA		
					body growth	no	NA		
					motor coordination (Rotarod)	no	NA		
					survival	no improvement: 53.5 days	–		
*NDUFS4*	*Ndufs4*^−/−^KO mouse	AAV2/9- *hNDUFS4*	CMV	2 × 10^12^ vg/mouse i.v. coupled with 3 × 10^11^ vg/mouse i.c.v. in P1	protein expression (WB)	↑ brain, heart, muscle	NA	no toxicity reported	Di Meo et al.^8^
					CI activity	↑ heart and muscle, brain	NA		
					body growth	↑	NA		
					motor coordination (Rotarod)	↑	NA		
					survival	prolonged to 82 days	–		
*NDUFS4*	*Ndufs4*^−/−^KO mouse	AAV-PHP.B-*hNDUFS4*	CMV	10^12^ or 2 × 10^12^ vg/mouse (pooled) i.v. in P26–28	protein expression (WB)	↑ brain	2 mpi	no toxicity reported	Silva-Pinheiro et al.^9^
					CI activity (BN)	↑ brain	2 mpi		
					body growth	↑	–		
					motor coordination (Rotarod)	↑	7–12 wpi		
					survival	prolonged to 100 days	–		
*NDUFS4*	*Ndufs4*^−/−^KO mouse	AAV-PHP.B-*hNDUFS4*	CMV	10^12^ vg/mouse i.v. in P1	protein expression (WB)	low ↑ brain	40 dpi	no toxicity reported	Silva-Pinheiro et al.^9^
					survival	no improvement	–		
*NDUFS4*	*Ndufs4*^−/−^KO mouse	AAV-PHP.B-*hNDUFS4*	CBA	10^12^ GC i.v. in P30	protein expression (WB)	↑ liver, heart, muscle, retina, and brain	15 dpi	no toxicity reported	Reynaud-Dulaurier et al.^10^
					CI activity (BN-IGA)	↑ brain	NA		
					body growth	↑	NA		
					motor coordination (cylinder and clasping tests)	↑	15 dpi		
					hypothermia	rescued	NA		
					survival	prolonged to 250 days	–		
*NDUFS4*	*Ndufs4*^−/−^KO mouse	scAAV9- *hNDUFS4*	CAG	10^11^ viral units i.v. in P1	protein expression (WB)	↑ brain	NA	no toxicity reported	Corrà et al.^11^
					CI activity (BN)	↑ brain	NA		
					body growth	↑	NA		
					motor coordination (Rotarod)	↑	70 dpi		
					survival	prolonged to 100 days	–		
*NDUFS4*	*Ndufs4*^−/−^KO mouse	scAAV9- *hNDUFS4*	CAG	double contemporary 10^11^ viral units i.v. and 10^10^ viral units i.c.v. in P1	protein expression (WB)	↑ brain	NA	no toxicity reported	Corrà et al.^11^
					CI activity (BN)	↑ brain	NA		
					body growth	↑	NA		
					motor coordination (Rotarod)	↑	NA		
					survival	prolonged to 270 days	–		
*NDUFS3*	smKO *Ndufs3*^−/−^mouse	rAAV9-*hNDUFS3*	CMV	1.25 × 10^15^ vg/kg retro-orbital in P15–18	protein expression (WB)	↑ muscle	15 mpi	no toxicity reported	Pereira et al.^12^
					CI activity (BN)	↑ muscle	13 mpi		
					myopathy (histology and SDHA staining)	recovered	15 mpi		
					body growth	↑	5 mpi		
					motor coordination (treadmill)	↑	5 mpi		
					survival	prolonged to 15 months	–		
*NDUFS3*	smKO *Ndufs3*^−/−^mouse	rAAV9-*hNDUFS3*	CMV	1.25 × 10^15^ vg/kg retro-orbital in P60	protein expression (WB)	↑ muscle	4 mpi	no toxicity reported	Pereira et al.^12^
					CI activity (BN)	↑ muscle	4 mpi		
					myopathy (histology and SDHA staining)	recovered	3 mpi		
					body weight	↑	3 mpi		
					motor coordination (treadmill)	↑	3 mpi		
*ND4*	R304H mouse	AAV2-*hND4*	HSP	1.08 × 10^11^ vg/mL intraocular	transgene expression (PCR)	↑ retina	NA	no toxicity reported	Yu et al.^13^
					visual function (PERG)	recovered	12 mpi		
					axonal density (TEM)	improvement	12 mpi		
*ND4*	R304H mouse	scAAV2- *hND4*	HSP	5.02 × 10^11^ particles/mL intraocular	transgene expression (confocal microscopy)	↑ retina	4 mpi	no toxicity reported and normal ocular histopathology in rodent and NHPs	Koilkonda et al.^14^
					visual function (PERG)	recovered	12 mpi		
					retinal structure (SD-OCT)	recovered	12 mpi		
*ND1*	patient’s fibroblasts m.3460G>A	allotopic gene expression	CMV	–	protein expression (WB)	↑	NA	–	Bonnet et al.^15^
					mitochondrial morphology (IF)	↑	NA		
					cell growth rate	↑	NA		
					CI activity (enzymatic assay)	↑	NA		
*ND4*	patient’s fibroblasts m.11778G>A	allotopic gene expression	CMV	–	protein expression (WB)	↑	NA	–	Bonnet et al.^15^
					mitochondrial morphology (IF)	↑	NA		
					cell growth rate	↑	NA		
					CI activity (enzymatic assay)	↑	NA		

**Isolated CIV deficiency**									

*SURF1*	*Surf1*^−/−^ KO mouse	scAAV9-*hSURF1*	CBh	2 × 10^11^ vg/mouse intrathecal in P30	protein expression (WB)	↑ brain	NA	no toxicity reported	Ling et al.^16^
					COX activity (spectrophotometry)	↑ liver	4 wpi		
					blood lactate	↓	10 mpi		
*SURF1*	*Surf1*^−/−^ KO mouse	scAAV9-*hSURF1*	CBh	8 × 10^11^ vg/mouse intrathecal in P30	protein expression (WB)	↑ brain	NA	no toxicity reported	Ling et al.^16^
					COX activity (spectrophotometry)	↑ brain and liver	4 wpi		
					blood lactate	↓	10 mpi		
*SURF1*	*Surf1*^−/−^ KO mouse	scAAV9-*hSURF1*	CBh	8 × 10^11^ vg/mouse intrathecal and 8 × 10^11^ vg/mouse i.v. in P30	protein expression (WB)	↑ brain	NA	no toxicity reported	Ling et al.^16^
					COX activity (spectrophotometry)	↑ brain and liver (4 wpi)	4 wpi		

**Isolated CV deficiency**									

*ATP6-8*	m.8529G>A cybrid cell line	allotopic gene expression	CMV	***–***	CV activity	↑	NA	***–***	Boominathan et al.^17^
					OCR (Seahorse)	↑	NA		
					cell viability	↑	NA		
*ATP6-8*	293T cells	allotopic gene expression	CMV	***–***	ATP synthesis	↑	NA	***–***	Manfredi et al.^18^
					cell growth	↑	NA		
*ATP6-8*	HeLa cells	allotopic gene expression	CMV	***–***	protein expression (WB)	↑	NA	***–***	Kaltimbacher et al.^19^
					mitochondrial localization (IF)	↑	NA		

**Defects in mitochondrial dynamics and translocators**									

*OPA1*	*Opa1*^delTTAG^	AAV2-h*OPA1*	CMV	1.6 × 10^11^ vg/mL intravitreal in P90	gene expression (qPCR)	↑ optic nerve and retina	2 mpi	no toxicity reported	Sarzi et al.^20^
					visual acuity (VEP and optokinetic drum)	comparable with WT	9 mpi		
					RGCs counting (Brn3a staining)	↑	8 mpi		
*OPA1*	DOA-iPSC (c.1334G>A, p.R445H)	CRISPR-Cas9	–	nucleofection	mitochondrial network morphology (TOMM20 staining)	restored	–	no toxicity reported	Sladen et al.^21^
					mitochondrial respiration (Seahorse)	↑	–		
*SLC25A46*	*Slc25a46*^−/−^ mouse	AAV-PHP.B-*Slc25a46*	CMV	1 × 10^11^ GC/g by face vein in P1	protein expression (WB)	↑ CNS	4 wpi	no toxicity reported	Yang et al.^22^
					body weight	no	2 mpi		
					cerebellar neuropathy (IF)	restored	40 dpi		
					mitochondrial network morphology (TEM)	restored	NA		
					complexes I–IV activity (spectrophotometry)	↑	NA		
					survival	prolonged to 100 days	–		
*SLC25A46*	*Slc25a46*^−/−^ mouse	AAV-PHP.B-*Slc25a46*	CMV	2 × 10^11^ GC/g by face vein in P1	protein expression (WB)	↑ brain	4 wpi	no toxicity reported	Yang et al.^22^
					body weight	↑	2 mpi		
					cerebellar neuropathy (IF)	restored	40 dpi		
					mitochondrial network morphology (TEM)	restored	NA		
					complexes I–IV activity (spectrophotometry)	↑	NA		
					survival	prolonged to 110 days	–		
*SLC25A4*	*Slc25a4*^−/−^ mouse	AAV2-*Ant1*	CMV	1 × 10^9^ infection units three times in the gastrocnemius in P1	protein expression (WB)	↑ gastrocnemius and liver	Up to 1 ypi	no toxicity reported	Flierl et al.^23^
					mitochondrial respiration	↑	Up to 1 ypi		
					myopathy (histological analysis)	recovered	2 wpi		

**Coenzyme Q10 deficiency**									

*COQ4*	patient-derived iPSCs (c.483G>C)	CRISPR-Cas9	–	Lipofectamine 3000	CoQ10 biosynthesis	↑	NA	no toxicity reported	Romero-Moya et al.^24^
					complexes activity (spectrophotometry)	↑ complexes I + III and II + III	NA		
					mitochondrial respiration (Seahorse)	↑ ATP production	NA		

**Multiple OXPHOS defects**									

*TYMP*	TP-deficient B lymphoblastoid cells	lentivirus-h*TYMP*	hPGK	*ex vivo* lentivirus transduction	protein expression (WB)	↑	NA	no cytotoxicity	Torres-Torronteras et al.^25^
					TP activity	↑	NA		
					Thd and dUrd concentration	↓	NA		
*TYMP*	*Tymp*^−/−^/*Upp*^−/−^mouse	HSCGT lentivirus-h*TYMP*	hPGK	infusion of transduced cells by tail vein after sublethal myeloablation	protein expression (WB)	↑ blood plasma	4 wpi	no toxicity reported	Torres-Torronteras et al.^25^
					TP activity (UPLC)	↑	29 wpi		
					Thd and dUrd concentration (UPLC)	↓ blood plasma	NA		
*TYMP*	*Tymp*^−/−^/*Upp*^−/−^mouse	HSCGT lentivirus-h*TYMP*	hPGK	5 × 10^15^ transduced cells by tail vein injection in 4–17-week mice	TP activity (UPLC)	↑ brain and small intestine	NA	no toxicity reported	Yadak et al.^26^
					Thd and dUrd concentration (UPLC or HPLC-MS)	↓ brain and small intestine	11 mpi		
					white matter vacuolation (MRI)	↓	6–12-month mice		
					astrocyte perivascular processes (IHC)	↓ thickness	6–12-month mice		
*TYMP*	*Tymp*^−/−^/*Upp*^−/−^mouse	AAV2/8- h*TYMP*	TBG	2 × 10^11^ GC/kg by i.v. in 8–12-week mice	protein expression (WB)	slight ↑ liver	NA	no toxicity reported; incidence of liver tumors comparable with WT and untreated KO	Torres-Torronteras et al.^27^^,^^28^
					TP activity (UPLC)	short-term: no	34 wpi		
						long-term: no	21 mpi		
					Thd and dUrd blood concentration (UPLC or LC-MS/MS)	short-term: ↓ to WT levels	28 wpi		
						long-term: = to untreated KO	88 wpi		
					Thd and dUrd tissues concentration (UPLC or LC-MS/MS)	short-term: ↓ liver, brain, and skeletal muscle	8 mpi		
						long-term: = to WT in liver	88 wpi		
					dNTP pool imbalance (polymerase-based assay)	short-term: no	8 mpi		
						long-term: no in liver	88 wpi		
					survival	no improvement	–		
*TYMP*	*Tymp*^−/−^/*Upp*^−/−^mouse	AAV2/8- h*TYMP*	TBG	10^12^ GC/kg by i.v. in 8–12-week mice	protein expression (WB)	↑ liver	NA	no toxicity reported; incidence of liver tumors comparable with WT and untreated KO	Torres-Torronteras et al.^27^^,^^28^
					TP activity (UPLC)	short-term: ↑ dose-dependent	34 wpi		
						long-term: ↑	21 mpi		
					Thd and dUrd blood concentration (UPLC or LC-MS/MS)	short-term: over ↓	28 wpi		
						long-term: ↓	88 wpi		
					Thd and dUrd tissues concentration (UPLC or LC-MS/MS)	short-term: ↓ liver, brain, and skeletal muscle	8 mpi		
						long-term: over ↓ in liver	88 wpi		
					dNTP pool imbalance (polymerase-based assay)	short-term: dose-dependent ↑ dCTP and dGTP	8 mpi		
						long-term: no in liver	88 wpi		
					survival	no improvement	–		
*TYMP*	*Tymp*^−/−^/*Upp*^−/−^mouse	AAV2/8- h*TYMP*	TBG	2 × 10^12^ GC/kg by i.v. in 8–12-week mice	protein expression (WB)	↑ liver	NA	No toxicity reported; incidence of liver tumors comparable with WT and untreated KO	Torres-Torronteras et al.^27^^,^^28^
					TP activity (UPLC)	short-term: ↑ dose-dependent	34 wpi		
						long-term: ↑	21 mpi		
					Thd and dUrd blood concentration (UPLC or LC-MS/MS)	short-term: over ↓	28 wpi		
						long-term: ↓	88 wpi		
					Thd and dUrd tissues concentration (UPLC or LC-MS/MS)	short-term: ↓ liver, brain, and skeletal muscle	8 mpi		
						long-term: over ↓ in liver	88 wpi		
					dNTP pool imbalance (polymerase-based assay)	short-term: dose-dependent ↑ dCTP and dGTP	8 mpi		
						long-term: ↓ dTTP ↑ dCTP	88 wpi		
					survival	no	–		
*TYMP*	*Tymp*^−/−^/*Upp*^−/−^mouse	AAV2/8- h*TYMP*	TBG	10^13^ GC/kg by i.v. in 8–12-week mice	protein expression (WB)	↑ liver	NA	no toxicity reported	Torres-Torronteras et al.^27^
					TP activity (UPLC)	↑ dose-dependent	34 wpi		
					Thd and dUrd blood concentration (UPLC or LC-MS/MS)	over↓	28 wpi		
					Thd and dUrd tissues concentration (UPLC or LC-MS/MS)	↓ liver, brain, and skeletal muscle	8 mpi		
					dNTP pool imbalance (polymerase-based assay)	dose-dependent ↑ dCTP and dGTP	8 mpi		
*TYMP*	Thd/dUrd-exposed *Tymp*^−/−^/*Upp1*^−/−^ mouse	AAV8- h*TYMP*	TBG	5 × 10^11^ vg/kg by i.v. in 8–11-week mice	TP activity (UPLC)	dose-dependent ↑	NA	no toxicity reported	Vila-Julià et al.^29^
					Thd and dUrd concentration (UPLC or LC-MS/MS)	↓ liver	24 wpi		
					leukoencephalopathy (MRI)	no	16 mpi		
					motor impairment (Rotarod)	no	14 wpi		
					dNTP pool imbalance (polymerase-based assay)	no significant amelioration	20 mpi		
*TYMP*	Thd/dUrd-exposed *Tymp*^−/−^/*Upp1*^−/−^ mouse	AAV8- h*TYMP*	TBG	10^12^ vg/kg by i.v. in 8–11-week mice	TP activity (UPLC)	dose-dependent ↑	NA	no toxicity reported	Vila-Julià et al.^29^
					Thd and dUrd concentration (UPLC or LC-MS/MS)	no significant ↓	24 wpi		
					leukoencephalopathy (MRI)	no	16 mpi		
					motor impairment (Rotarod)	no	14 wpi		
					dNTP pool imbalance (polymerase-based assay)	no significant amelioration	20 mpi		
*TYMP*	Thd/dUrd-exposed *Tymp*^−/−^/*Upp1*^−/−^ mouse	AAV8- h*TYMP*	TBG	2 × 10^12^ vg/kg by i.v. in 8–11-week mice	TP activity (UPLC)	dose-dependent ↑	NA	no toxicity reported	Vila-Julià et al.^29^
					Thd and dUrd concentration (UPLC or LC-MS/MS)	↓ liver	24 wpi		
					leukoencephalopathy (MRI)	no	16 mpi		
					motor impairment (Rotarod)	no	14 wpi		
					dNTP pool imbalance (polymerase-based assay)	no significant amelioration	20 mpi		
*TYMP*	Thd/dUrd-exposed *Tymp*^−/−^/*Upp1*^−/−^ mouse	AAV8- h*TYMP*	TBG	10^13^ vg/kg by i.v. in 8–11-week mice	TP activity (UPLC)	dose-dependent ↑	NA	no toxicity reported	Vila-Julià et al.^29^
					Thd and dUrd concentration (UPLC or LC-MS/MS)	not significant ↓	–		
					leukoencephalopathy (MRI)	no rescue	16 mpi		
					motor impairment (Rotarod)	no	14 wpi		
					dNTP pool imbalance (polymerase-based assay)	↓ dTTP in liver	20 mpi		
*TYMP*	Thd/dUrd-exposed *Tymp*^−/−^/*Upp1*^−/−^ mouse	AAV8- h*TYMP*	AAT	2 × 10^12^ vg/kg by i.v. in 8–11-week mice	TP activity (UPLC)	dose-dependent ↑	NA	no toxicity reported	Vila-Julià et al.^29^
					Thd and dUrd concentration (UPLC or LC-MS/MS)	↓ blood, liver, and brain	24 wpi		
					leukoencephalopathy (MRI)	↓	16 mpi		
					motor impairment (Rotarod)	No	14 wpi		
					dNTP pool imbalance (polymerase-based assay)	↓ dTTP in brain; ↑ dCTP in liver	20 mpi		
*TYMP*	Thd/dUrd-exposed *Tymp*^−/−^/*Upp1*^−/−^ mouse	AAV8- h*TYMP*	AAT	10^13^ vg/kg by i.v. in 8–11-week mice	TP activity (UPLC)	dose-dependent ↑	NA	no toxicity reported	Vila-Julià et al.^29^
					Thd and dUrd concentration (UPLC or LC-MS/MS)	↓ liver	24 wpi		
					leukoencephalopathy (MRI)	↓	16 mpi		
					motor impairment (Rotarod)	no	14 wpi		
					dNTP pool imbalance (polymerase-based assay)	↑ dCTP in liver	20 mpi		
*TYMP*	Thd/dUrd-exposed *Tymp*^−/−^/*Upp1*^−/−^ mouse	AAV8- h*TYMP*	HLP	2 × 10^12^ vg/kg by i.v. in 8–11-week mice	TP activity (UPLC)	dose-dependent ↑	NA	no toxicity reported	Vila-Julià et al.^29^
					Thd and dUrd concentration (UPLC or LC-MS/MS)	↓ liver	24 wpi		
					leukoencephalopathy (MRI)	↓	16 mpi		
					motor impairment (Rotarod)	no	14 wpi		
					dNTP pool imbalance (polymerase-based assay)	↑ dCTP in liver	20 mpi		
*TYMP*	Thd/dUrd-exposed *Tymp*^−/−^/*Upp1*^−/−^ mouse	AAV8- h*TYMP*	HLP	10^13^ vg/kg by i.v. in 8–11-week mice	TP activity (UPLC)	dose-dependent ↑	NA	no toxicity reported	Vila-Julià et al.^29^
					Thd and dUrd concentration (UPLC or LC-MS/MS)	↓ liver	24 wpi		
					leukoencephalopathy (MRI)	↓	16 mpi		
					motor impairment (Rotarod)	no	14 wpi		
					dNTP pool imbalance (polymerase-based assay)	no significant amelioration	20 mpi		
*TYMP*	*Tymp*^−/−^/*Upp1*^−/−^ mouse	AAV2/8- h*TYMP*	TBG	5 × 10^10^ vg/kg by i.v. in 8–12-week mice	protein expression (WB)	no ↑	8 mpi	no toxicity reported	Cabrera-Pérez et al.^30^
					TP activity (UPLC)	↑ liver	8 mpi		
					Thd and dUrd concentration (UPLC or LC-MS/MS)	small ↓ blood plasma and liver	24 wpi		
					dNTP pool imbalance (polymerase-based assay)	no	NA		
*TYMP*	*Tymp*^−/−^/*Upp1*^−/−^ mouse	AAV2/8- h*TYMP*	TBG	2 × 10^11^ vg/kg by i.v. in 8–12-week mice	protein expression (WB)	↑ liver	8 mpi	only small and transient increase of plasma ALT activity	Dalla Rosa et al.^31^
					TP activity (UPLC)	↑ liver	8 mpi		
					Thd and dUrd concentration (UPLC or LC-MS/MS)	↓ blood plasma, liver, brain, skeletal muscle, and small intestine	24 wpi		
					dNTP pool imbalance (polymerase-based assay)	↓ dTTP; ↑ dCTP	NA		
*TYMP*	*Tymp*^−/−^/*Upp1*^−/−^ mouse	AAV2/8- h*TYMP*	TBG	5 × 10^11^ vg/kg by i.v. in 8–12-week mice	protein expression (WB)	↑ liver	8 mpi	no toxicity reported	Cabrera-Pérez et al.^30^
					TP activity (UPLC)	↑ liver	8 mpi		
					Thd and dUrd concentration (UPLC or LC-MS/MS)	↓ blood plasma, liver, brain, and skeletal muscle	24 wpi		
					dNTP pool imbalance (polymerase-based assay)	↓ dTTP; ↑ dCTP; ↑ dGTP	NA		
*TYMP*	*Tymp*^−/−^/*Upp1*^−/−^ mouse	AAV2/8- h*TYMP*	PKG	2 × 10^11^ vg/kg by i.v. in 8–12-week mice	protein expression (WB)	no	8 mpi	only small and transient increase of plasma ALT activity	Cabrera-Pérez et al.^30^
					TP activity (UPLC)	no	8 mpi		
					Thd and dUrd concentration (UPLC or LC-MS/MS)	small ↓ blood plasma and liver	24 wpi		
					dNTP pool imbalance (polymerase-based assay)	↓ dTTP	NA		
*TYMP*	*Tymp*^−/−^/*Upp1*^−/−^ mouse	AAV2/8- h*TYMP*	PKG	5 × 10^11^ vg/kg by i.v. in 8–12-week mice	Protein expression (WB)	No	8 mpi	no toxicity reported	Cabrera-Pérez et al.^30^
					TP activity (UPLC)	No	8 mpi		
					Thd and dUrd concentration (UPLC or LC-MS/MS)	↓ blood plasma, liver, brain, skeletal muscle, and small intestine	24 wpi		
					dNTP pool imbalance (polymerase-based assay)	↓ dTTP; ↑ dCTP	NA		
*TYMP*	*Tymp*^−/−^/*Upp1*^−/−^ mouse	AAV2/8- h*TYMP*	PKG	10^12^ vg/kg by i.v. in 8–12-week mice	protein expression (WB)	small ↑ liver	8 mpi	no toxicity reported	Cabrera-Pérez et al.^30^
					TP activity (UPLC)	no	8 mpi		
					Thd and dUrd concentration (UPLC or LC-MS/MS)	↓ blood plasma, liver, brain, skeletal muscle, and small intestine	24 wpi		
					dNTP pool imbalance (polymerase-based assay)	↓ dTTP; ↑ dCTP	NA		
*TYMP*	*Tymp*^−/−^/*Upp1*^−/−^ mouse	AAV2/8- h*TYMP*	PKG	2 × 10^12^ vg/kg by i.v. in 8–12-week mice	protein expression (WB)	small ↑ liver	8 mpi	no toxicity reported	Cabrera-Pérez et al.^30^
					TP activity (UPLC)	no	8 mpi		
					Thd and dUrd concentration (UPLC or LC-MS/MS)	↓ blood plasma, liver, brain, and skeletal muscle	24 wpi		
					dNTP pool imbalance (polymerase-based assay)	↓ dTTP; ↑ dCTP	NA		
*TYMP*	*Tymp*^−/−^/*Upp1*^−/−^ mouse	scAAV2/8- h*TYMP*	HLP	2 × 10^11^ vg/kg by i.v. in 8–12-week mice	protein expression (WB)	small ↑ liver	8 mpi	no toxicity reported	Cabrera-Pérez et al.^30^
					TP activity (UPLC)	no	8 mpi		
					Thd and dUrd concentration (UPLC or LC-MS/MS)	↓ blood plasma, liver, brain, skeletal muscle, and small intestine	24 wpi		
					dNTP pool imbalance (polymerase-based assay)	↓ dTTP	NA		
*TYMP*	*Tymp*^−/−^/*Upp1*^−/−^ mouse	scAAV2/8- h*TYMP*	HLP	5 × 10^11^ vg/kg by i.v. in 8–12-week mice	protein expression (WB)	small ↑ liver	8 mpi	no toxicity reported	Cabrera-Pérez et al.^30^
					TP activity (UPLC)	no	8 mpi		
					Thd and dUrd concentration (UPLC or LC-MS/MS)	↓ blood plasma, liver, brain, skeletal muscle, and small intestine	24 wpi		
					dNTP pool imbalance (polymerase-based assay)	↓ dTTP; ↑ dCTP	NA		
*TYMP*	*Tymp*^−/−^/*Upp1*^−/−^ mouse	scAAV2/8- h*TYMP*	HLP	10^12^ vg/kg by i.v. in 8–12-week mice	protein expression (WB)	↑ liver	8 mpi	no toxicity reported	Cabrera-Pérez et al.^30^
					TP activity (UPLC)	no	8 mpi		
					Thd and dUrd concentration (UPLC or LC-MS/MS)	↓ blood plasma, liver, brain, skeletal muscle, and small intestine	24 wpi		
					dNTP pool imbalance (polymerase-based assay)	↓ dTTP; ↑ dCTP	NA		
*TYMP*	*Tymp*^−/−^/*Upp1*^−/−^ mouse	scAAV2/8- h*TYMP*	HLP	2 × 10^12^ vg/kg by i.v. in 8–12-week mice	protein expression (WB)	↑ liver	8 mpi	no toxicity reported	Cabrera-Pérez et al.^30^
					TP activity (UPLC)	no	8 mpi		
					Thd and dUrd concentration (UPLC or LC-MS/MS)	↓ blood plasma, liver, brain, skeletal muscle, and small intestine	24 wpi		
					dNTP pool imbalance (polymerase-based assay)	↓ dTTP; ↑ dCTP	NA		
*TYMP*	*Tymp*^−/−^/*Upp1*^−/−^ mouse	AAV2/8- h*TYMP*	AAT	5 × 10^10^ vg/kg by i.v. in 8–11-week mice	protein expression (WB)	↑ liver	8 mpi	no toxicity reported	Cabrera-Pérez et al.^30^
					TP activity (UPLC)	↑ liver	8 mpi		
					Thd and dUrd concentration (UPLC or LC-MS/MS)	↓ blood plasma, liver, brain, skeletal muscle, and small intestine	24 wpi		
					dNTP pool imbalance (polymerase-based assay)	↓ dTTP; ↓ dGTP	NA		
*TYMP*	*Tymp*^−/−^/*Upp1*^−/−^ mouse	AAV2/8- h*TYMP*	AAT	2 × 10^11^ vg/kg by i.v. in 8–11-week mice	protein expression (WB)	↑ liver	8 mpi	no toxicity reported	Cabrera-Pérez et al.^30^
					TP activity (UPLC)	↑ liver	8 mpi		
					Thd and dUrd concentration (UPLC or LC-MS/MS)	↓ blood plasma, liver, brain, skeletal muscle, and small intestine	24 wpi		
					dNTP pool imbalance (polymerase-based assay)	↓ dTTP; ↑ dCTP	NA		
*TYMP*	*Tymp*^−/−^/*Upp1*^−/−^ mouse	AAV2/8- h*TYMP*	AAT	5 × 10^11^ vg/kg by i.v. in 8–11-week mice	protein expression (WB)	↑ liver and brain	8 mpi	no toxicity reported	Cabrera-Pérez et al.^30^
					TP activity (UPLC)	↑ liver	8 mpi		
					Thd and dUrd concentration (UPLC or LC-MS/MS)	↓ blood plasma, liver, brain, skeletal muscle, and small intestine	24 wpi		
					dNTP pool imbalance (polymerase-based assay)	↓ dTTP; ↓ dCTP; ↓ dGTP	NA		
*TYMP*	*Tymp*^−/−^/*Upp1*^−/−^ mouse	AAV2/8- h*TYMP*	AAT	10^12^ vg/kg by i.v. in 8–11-week mice	protein expression (WB)	↑ liver and brain	8 mpi	no toxicity reported	Cabrera-Pérez et al.^30^
					TP activity (UPLC)	↑ liver	8 mpi		
					Thd and dUrd concentration (UPLC or LC-MS/MS)	↓ blood plasma, liver, brain, skeletal muscle, and small intestine	24 wpi		
					dNTP pool imbalance (polymerase-based assay)	↓ dTTP; ↓ dCTP; ↓ dGTP	NA		
*TYMP*	*Tymp*^−/−^/*Upp1*^−/−^ mouse	AAV2/8- h*TYMP*	AAT	2 × 10^12^ vg/kg by i.v. in 8–11-week mice	protein expression (WB)	↑ liver, brain, and small intestine	8 mpi	no toxicity reported	Cabrera-Pérez et al.^30^
					TP activity (UPLC)	↑ liver and brain	8 mpi		
					Thd and dUrd concentration (UPLC or LC-MS/MS)	↓ blood plasma, liver, brain, skeletal muscle, and small intestine	24 wpi		
					dNTP pool imbalance (polymerase-based assay)	↓ dTTP; ↓ dCTP; ↓ dGTP	NA		
*MPV17*	*Mpv17*^−/−^ mouse	AAV2/8-*hMPV17*	TGB	4 × 10^32^ vg/kg by retro-orbital injection in P60	protein expression (WB)	↑ liver	3 wpi	no toxicity reported	Bottani et al.^33^
					mtDNA levels	↑	3 wpi		
					CI–CIV activity (spectrophotometry)	↑ CI-III-IV	3 wpi		
					liver damage (histology)	prevented	3 wpi		
					blood transaminases	↓	3 wpi		
*DGUOK*	*dGUOK*^−/−^ mouse	AAV9- hDGUOK	CAG	8 × 10^13^ vg/kg i.v. in P2	protein expression (WB)	↑ liver	NA	no toxicity reported	Keshavan et al.^81^
					mtDNA levels	dose-dependent ↑ liver and skeletal muscle	NA		
					CI–IV activity (spectrophotometry)	↑ CI-III-IV in liver; ↑ CI in skeletal muscle	NA		
					blood ALT, AST, ALP	↓ ALT, partial ↓ AST and ALP	NA		
					body growth	only in females	NA		
					survival	no significant improvement: 261 days	NA		
*DGUOK*	*dGUOK*^−/−^ mouse	AAV9- hDGUOK	CAG	8 × 10^14^ vg/kg i.v. in P2	protein expression (WB)	↑ liver	NA	decrease in growth and a single case of hepatocellular carcinoma	Keshavan et al.^81^
					mtDNA levels	dose-dependent ↑ liver and skeletal muscle	NA		
					CI–IV activity (spectrophotometry)	↑ CI-III-IV in liver; ↑ CI in skeletal muscle	NA		
					blood ALT, AST, ALP	↓ ALT, partial ↓ AST and ALP	NA		
					body growth	only in females	NA		
					survival	significant improvement: 42 weeks	–		
*DGUOK*	*dGUOK*^−/−^ mouse	AAV9- hDGUOK	CAG	8 × 10^15^ vg/kg i.v. in P2	survival	no improvement: 20 days	–	toxicity caused early death	Keshavan et al.^81^
*TK2*	*TK2* H126N KI mouse	AAV9-*hTK2*	CBA	4 × 10^10^ vg i.v. in P1	gene expression (qPCR)	↑ liver, brain, and skeletal muscle	60 dpi	renal dysfunction	Lopez-Gomez et al.^35^
					mtDNA levels	short-term: ↑ liver, brain, skeletal muscle; mild ↑ kidneys	29 dpi		
						long term: mild ↓ liver, brain, skeletal muscle; severe ↓ kidneys	60 dpi		
					TK2 activity (tritium-labeled bromovinyl deoxyuridine)	short-term: ↑ liver, brain, skeletal muscle	29 dpi		
						long term: ↑ brain, skeletal muscle; stable in liver	60 dpi		
					body growth	↑, then weakness required euthanasia	20 dpi		
					survival	39 days	–		
*TK2*	*TK2* H126N KI mouse	AAV9-*hTK2*	CBA	4 × 10^11^ vg i.v. in P1	gene expression (qPCR)	↑ liver, brain, and skeletal muscle	60 dpi	renal dysfunction	Lopez-Gomez et al.^35^
					mtDNA levels	short-term: ↑ liver, brain, skeletal muscle; mild ↑ kidneys	29 dpi		
						long-term: mild ↓ liver, brain, skeletal muscle; severe ↓ kidneys	60 dpi		
					TK2 activity (tritium-labeled bromovinyl deoxyuridine)	short-term: ↑ liver, brain, skeletal muscle	29 dpi		
						long-term: ↑ brain, skeletal muscle; stable in liver	60 dpi		
					body growth	↑	140 dpi		
					survival	prolonged to 88.5 days	–		
*TK2*	*TK2* H126N KI mouse	AAV9-*hTK2/*AAV2-*hTK2*	CBA	2.1 × 10^11^ vg AAV9 i.v. in P1 and 1.05 × 10^11^ vg AAV2 by i.v. in P29	gene expression (qPCR)	↑ liver, brain, and skeletal muscle	60 dpi	partial amelioration of renal dysfunction	Lopez-Gomez et al.^35^
					mtDNA levels	mild ↓ liver, brain, skeletal muscle	60 dpi		
					TK2 activity (tritium-labeled bromovinyl deoxyuridine)	↑ brain, skeletal muscle; stable in liver; severe ↓ kidneys	60 dpi		
					body growth	↑	180 dpi		
					survival	prolonged to 120 days	–		
*TK2*	*TK2* H126N KI mouse	AAV9-*hTK2/*AAV2-*hTK2*	CBA	2.1 × 10^11^ vg AAV9 i.v. in P1 and 1.05 × 10^11^ vg AAV2 by i.v. in P29 + co-treatment with 520 mg/kg/day each of dC and dT from PD21	gene expression (qPCR)	↑ liver, brain, and skeletal muscle	60 dpi	no toxicity reported	Lopez-Gomez et al.^35^
					mtDNA levels	↑ liver; mild ↑ kidneys; mild ↓ brain, skeletal muscle	60 dpi		
					TK2 activity (tritium-labeled bromovinyl deoxyuridine)	↑ brain, skeletal muscle; ↓ liver, kidneys	60 dpi		
					body growth	↑	180 dpi		
					survival	prolonged to 181 days	–		

ARMS-qPCR, amplification-refractory mutation system quantitative PCR; BN, blue native; BN-IGA, blue native in-gel activity; dpi, days post-injection; KO, knockout; HPLC, high-performance liquid chromatography; IF, immunofluorescence; IHC, immunohistochemistry; i.v., intravenous; LC-MS/MS, liquid chromatography-mass spectrometry; mpi, months post-injection; MRI, magnetic resonance imaging; NA, not available; OCR, oxygen consumption rate; PERG, pattern electroretinogram; RGCs, retinal ganglion cells; SD-OCT, spectral domain optical coherence tomography; TEM, transmission electron microscopy; UPLC, ultra performance liquid chromatography; VEP, visual evoked potential; WB, western blot; wpi, weeks post-injection.

Another important molecular carrier is *SLC25A4*, also called *ANT1*, which encodes an inner membrane protein involved in the transport of ADP from the cytoplasm into the mitochondrial matrix and ATP in an inverse sense. Mutations in this gene are responsible for cardiomyopathy (MIM: 617184; MIM: 615418) and autosomal dominant progressive external ophthalmoplegia (MIM: 609283).

The *Slc25a4*^−/−^ mouse model^44^ was used to test the safety and efficacy of AAV2, charged with the mouse *Slc25a4* cDNA, transcribed by the CMV promoter (Tables 1 and 2). Animals were treated at PD1 with three separate injections of 2 μL of vector, for a total of 1 × 10^9^ infection units in the posterior group of muscles of one leg. The *Slc25a4* cDNA was found in muscle and partially in the liver, indicating a sign of systemic translocation, and gene expression was maintained up to 1 year of age. Protein levels changed from undetectable to 5%–30% and ATP transport improved by 45%. Histopathological analyses highlighted a great reduction in ragged red fibers (RRFs) and inflammation in the soleus and gastrocnemius muscles. The pre-clinical study was successful in terms of efficacy, with no severe adverse effects and a general improvement of the muscular condition of *Slc25a4*^−/−^ mice.^23^

### Coenzyme Q_10_ deficiency

Coenzyme Q_10_ shuttles electrons from CI (NADH ubiquinone oxidoreductase) and CII (succinate dehydrogenase) and electron-transferring flavoprotein dehydrogenase to CIII (ubiquinol cytochrome *c* reductase) in the inner mitochondrial membrane. Pathogenic variants in CoQ_10_ are causative for a spectrum of diseases, called CoQ_10_ primary deficiency (MIM: 607426), which include encephalopathy, severe infantile multisystemic disease, cerebellar ataxia, isolated myopathy, and nephrotic syndrome.^82^^,^^83^ Biochemically, a combined deficiency in CI + CIII and/or CII + CIII is present in patients’ biological samples. Supplementation with high doses of CoQ_10_ is the only available treatment for patients so far, although it presents poor CNS efficacy. Gene therapy has only been tested in iPSCs derived from skin fibroblasts of a 4-year-old girl presenting a mutational variant in the CoQ_4_ gene (c.483G>C; E161D). Untreated fibroblasts showed a reduction in both activity and biosynthetic levels of COQ_10_ compared with controls. To correct the mutant variant, authors use the CRISPR-Cas9 technology by nucleofecting iPSCs with sgRNA and a 90 bp-long donor ssDNA synthesized with the corrected codon. Homozygous clones displayed a 25% increase in CoQ_10_ concentration and a 50% augmentation of CoQ_10_ biosynthesis compared with unedited iPSCs. A 50-fold increase in the activity of CI + CIII and CII + CIII was also reported.^24^

### Multiple OXPHOS deficiencies

The synthesis of mtDNA requires replisome machinery, a balanced supply of nucleotides, as well as appropriate mitochondrial fusion to exchange intramitochondrial contents. The DNA replisome is composed of nuclear-encoded proteins,^84^ with the major role of the replicative mtDNA helicase Twinkle, DNA polymerase γ (Pol γ), and the mitochondrial single-stranded DNA-binding protein (mtSSB). The mtDNA polymerase γ (POLγ) is a heterotrimer composed of one catalytic subunit (POLγA) encoded by *POLG*, and a homodimeric accessory subunit (POLγB), encoded by *POLG2*, that binds asymmetrically to POLγ to increase the affinity between POLγ and mtDNA.^85^ Replication takes place after the unwinding of the dsDNA acted by the mtDNA helicases Twinkle and the annealing of the RNA primer in concomitance with the positioning of mitochondrial transcription factor A, encoded by *TFAM*.^86^ Mitochondrial deoxynucleotide triphosphates, building blocks of mtDNA replication, are produced as a result of either the *de novo* pathway, which operates in the cytosol, or the salvage pathway, which operates both in the cytosol and in mitochondria by recycling existing nucleotides from the diet or nucleic acid catabolism. Defects in enzymes involved in these pathways are the cause of depletion and imbalance of mitochondrial nucleotides, resulting in impairment of mtDNA synthesis.^87^ Gene therapy approaches have been investigated for defects of deoxynucleotides imbalance, such as thymidine phosphorylase deficiency causing mitochondrial neuro gastrointestinal encephalomyopathy (MNGIE) (*TYMP* gene; MIM: 603041), thymidine kinase 2 deficiency causing infantile, pediatric or adult myopathy (*TK2* gene; MIM: 609560), and MPV17 defects causing Navajo neuro-hepatopathy or hepatocerebral syndrome (*MPV17* gene; MIM: 618400; MIM: 256810) as detailed in the following session.

#### TYMP

TYMP encodes the enzyme thymidine phosphorylase (TP) responsible for the reversible phosphorolysis of thymidine (also called deoxythymidine) and deoxyuridine to 2-deoxyribose-1-phosphate, thymine, and uracil.^42^ Mutation in *TYMP* results in a deficiency of the enzymatic activity with consequent toxic excess of deoxythymidine and deoxyuridine. Mitochondrial DNA presents quantitative (depletion) and qualitative defects (multiple deletions and point mutations).^88^ Autosomal recessive variants in the *TYMP* gene cause MNGIE, characterized by progressive gastrointestinal dysmotility, cachexia, progressive external ophthalmoplegia, ptosis, leukoencephalopathy, and demyelinating peripheral sensory-motor neuropathy.^89^ Disease onset is between the second and fourth decades of life, and it rapidly progresses to death.^90^

Unlike humans, who rely solely on TP to catalyze the phosphorolysis of both thymidine and deoxyuridine, mice require an additional enzyme, the uridine phosphorylase (UPP), for dUrd phosphorolysis. UPP consists of two isoenzymes, uridine phosphorylase 1 (Upp1), which is broadly expressed, and uridine phosphorylase 2 (Upp2), which is limited to specific tissues such as the liver. To reproduce the human disease, a double knockout mouse for TP and uridine phosphorylase (Upp1) was generated.^45^

Details on the *Tymp*^−/−^/*Upp1*^−/−^ murine model and the use of exogenous dThd and dUrd stressing conditions for exacerbating the phenotype in the characterization of the animal model and specific studies for gene therapy are reported in Tables 1 and 2.^45^^,^^46^

Lentivirus-mediated gene therapy was attempted with the transduction of lymphoblastoid cells from two patients with a lentiviral vector containing a human copy of *TYMP* cDNA. Preliminary *in vitro* studies confirmed that the therapeutic vector successfully drove the expression of TP in the transduced cells. TP activity was detected, reaching a stable value of 5,000 nmol thymine h^−1^ per mg protein at week 28 after transduction. Ten and 20 μM of exogenous dThd and dUrd, which mimic the extracellular concentrations found in MNGIE patients, were added to the cell culture medium, and it demonstrated the ability of edited cells to eliminate the excess of nucleosides.^91^ *TYMP* lentivirus-mediated gene therapy under the control of PGK promoter was then tested in the *Tymp*^−/−^/*Upp1*^−/−^ murine model^45^ by using immunoselected hematopoietic lineage negative cells from double KO mice transduced with lentiviral vector at 100 MOI and then infused into partially myeloablated syngenic mice. Treated animals showed plasma levels of dThd and dUrd comparable with WT. Twenty-nine weeks after transplantation, all treated mice maintained a 20-fold higher TP activity than WT TP activity without impact on cellular differentiation.^25^ A few years later, Yadak et al. modified the previous strategy using a third-generation self-inactivating lentivirus containing a similar backbone, with an hPGK promoter and the re-coded human TYMP sequence (TPco) to enhance protein production with a reduced number of transduced donor cells and vector copy number. With this lentiviral vector-based hematopoietic stem cell gene therapy (HSCGT), treated animals displayed TP enzyme activity in the brain and small intestine and reduced nucleoside levels in skeletal muscle and liver. Through brain MRI, the white matter vacuolization in the cerebellar white matter and corpus callosum found in untreated animals was reversed by the treatment. Moreover, the impaired morphology of brain astrocytes was recovered in treated animals.^26^

The therapeutic effect was sustained over time but associated with a reduced lifespan in some animals. To address this limitation and to mitigate the risk of possible oncogenesis caused by the insertion of the lentiviral vector in the recipient DNA, Torres-Torronteras et al. explored an alternative strategy. Since AAV-mediated expression of the therapeutic gene in the liver represents a promising therapeutic approach for genetic metabolic disorders caused by systemic accumulation of toxic metabolites, as MNGIE, the authors chose the AAV2/8 serotype loaded with the human *TYMP* coding sequence activated by the liver-specific promoter thyroxine-binding globulin (TBG). Gene therapy was administered at PD16 with four different viral doses (2 × 10^11^, 10^12^, 2 × 10^12^, and 10^13^ GC/kg) in the *Tymp*^−/−^/*Upp1*^−/−^ murine model.^25^ Steady-state levels of TP protein and TP activity in the liver increased in treated animals in a dose-dependent manner. Plasma levels of dThd of 75% of mice treated with the lowest dose were stably decreased, and in 37.5% were maintained until 28 weeks of monitoring; mice treated with the higher doses showed an over-reduction below WT levels in almost all animals that were sustained over the entire time monitored. Eight months after the treatment, nucleosides were measured in the liver, brain, and skeletal muscle, and reduced levels mirrored the previous results, while only a nonsignificant tendency to decrease was found in the small intestine. AAV2/8-TBG-hcTYMP targeting the liver achieved the same biochemical efficacy as the lentiviral-based approach but without any negative impact on the lifespan or additional side effects.^27^^,^^28^ A different study from Cabrera-Pérez et al. explored the effect of various liver-specific promoters (TBG, HLP, and AAT), as well as a constitutive phosphoglycerate kinase promoter (PGK) for the expression of the *TYMP* transgene in the AAV8 serotype, using either single-stranded or self-complementary DNA. The authors treated double KO mice, aged 8–12 weeks, with a single i.v. tail injection, administering different doses ranging from 5 × 10^10^ to 2 × 10^12^ vg/kg. The vector with liver-specific promoters confirmed its ability to reestablish liver TP activity in extrahepatic tissues and normalized nucleoside homeostasis more effectively than the PGK promoter. Among the liver-specific promoters, the AAV-AAT construct showed the most significant efficacy. At 34 weeks after administration, plasma dThd levels were comparable with or below the WT level in 65% of animals treated with AAV-PGK, 83% in animals treated with AAV-TBG, 94% in animals treated with scAAV-HLP, and 97% in animals treated with AAV-AAT. Furthermore, the authors found no difference between the self-complementary DNA and the single-stranded configurations.^30^ Vila-Julia et al. experimented the AAV8 serotype to drive the human wild-type cDNA *TYMP* expression under different promoters: TBG, hybrid liver-specific (HLP), or α-1-antitrypsin (AAT) in a dose ranging from 5 × 10^11^ to 10^3^ vg/kg. For their experiment, the authors used the Thd/dUrd-exposed *Tymp*^−/−^/*Upp1*^−/−^ mouse,^46^ which was stressed with exogenous administration of thymidine (68.6 mM) and deoxyuridine (72.3 mM) at a standard concentration of 16.6 mg/mL in drinking water up to 2 years to exacerbate the pathological phenotype (Table 1).^46^ A single i.v. administration was performed via the tail vein at 8–11 weeks of age. A vector dose response was observed in most of the cases, and AAV-AAT resulted as the most effective in reducing dThd plasma concentration at 3.5 μM with the 10^3^ vg/kg dose, in line with the ability to restore TP activity in the liver. Both dThd and dUrd were found to be reduced in 22-month-old treated mice. A positive effect was also found in the rotarod test, in which 69% of AAV-treated mice had time to fall comparable with WT mice. Of relevance, this study unveiled that liver-targeted AAV-mediated gene therapy can also ameliorate the neurological phenotype observed in Thd/dUrd-exposed *Tymp*^−/−^/*Upp1*^−/−^ mice, and specifically the brain MRI T2-weighted hyperintense areas in *Tymp*^−/−^/*Upp1*^−/−^ mice. In contrast, AAV-TBG treatment showed no improvement in most cases.^29^

A key conclusion drawn from the various gene therapy strategies in MNGIE is that, although TP is expressed in multiple tissues, liver-targeted gene therapy has proven to be the most effective. This efficacy is attributed to the fact that MNGIE is caused by the toxic accumulation of Thd and dUrd, and hepatic TP expression alone appears sufficient to mediate the systemic clearance.

#### MPV17

MPV17 encodes for an inner mitochondrial channel-forming protein and is deemed to be involved in the maintenance of the mitochondrial deoxyribonucleotide pool, although its function is not completely characterized.^31^ Autosomal recessive variants in *MPV17* cause infantile onset of hepatocerebral disease with mtDNA depletion or Navajo neuro-hepatopathy with multiple mtDNA deletions in the liver. A late-onset variant with deletions in muscle associated with neuropathy and myopathy has also been described in a few cases.^92^^,^^93^

The AAV2/8 viral vector was exploited to deliver human *MPV17* cDNA in the liver controlled under a TBG promoter (TBG-AAV2/8-h*MPV17*) in *Mpv17*^−/−^mouse (Table 1).^47^ Mice were injected retro-orbitally at a concentration of 4 × 10^12^ GC/kg at PD60. The exclusive expression of the vector into the liver was confirmed by PCR. *MPV17* mRNA and protein levels, together with mtDNA copy number, were increased in *Mpv17*^−/−^ mice liver.^33^

***DGUOK***

Deoxyguanosine kinase (dGk), encoded by the nuclear gene *DGUOK*, is an enzyme of the salvage pathway that catalyzes the phosphorylation of deoxyguanosine and deoxyadenosine into their corresponding monophosphates. Autosomal recessive pathogenetic variants in the *DGUOK* gene cause dGk deficiency, one of the most common causes of hepatocerebral mtDNA depletion syndrome (MIM: 601465). Deoxyguanosine kinase deficiency was initially described as early-onset isolated hepatopathy with mtDNA depletion or myopathy with mtDNA multiple deletions.^87^^,^^94^^,^^95^ However, in our recent retrospective natural history study, including novel cases and literature review data, we clearly defined the disease as a tissue-specific mtDNA maintenance defect. Based on clinical and molecular findings, we recognized four major clinical forms: hepatocerebral, hepatomyocerebral, isolated hepatopathy, and isolated myopathy, presenting as a continuum spectrum from the neonatal to the adult onset.^96^

Keshavan et al. developed an AAV9-based gene therapy under the control of a CAG promoter and administered it i.v. via the temporal vein at P2, at two dosages: 8 × 10^13^ and 8 × 10^14^ vg/kg in *Dguok*^−/−^ mice (Table 1). AAV9 mediated efficient and sustained liver transduction, resulting in a dose-dependent increase in mtDNA copy number, with a significant increase of up to 82% at the higher dose. ALT levels were normalized, while only a partial recovery of AST and ALP was observed. Skeletal muscle also showed an increase in the mtDNA copy number. However, mtDNA depletion and isolated CIV deficiency remained unresolved in the brain, indicating limited brain transduction of both vectors. Growth improvement was observed in females but not in males, and WT treated with the higher dose exhibited growth impairment. Locomotor activity was not rescued. Survival was not significantly improved at the lower dose but showed a substantial increase at the higher dose. Notably, one KO mouse developed hepatocellular carcinoma following the 8 × 10^14^ vg/kg treatment, raising concerns about the potential toxicity of the higher dose.^81^

#### TK2

TK2 encodes for thymidine kinase 2, an enzyme that phosphorylates deoxycytidine and thymidine to generate the corresponding monophosphates. Autosomal recessive variants in the *TK2* gene cause myopathy classified in three main clinical forms: infantile TK2d with onset before 1 year of age and mtDNA depletion; childhood TK2d with onset between 1 and 12 years of age and mtDNA depletion and/or mtDNA deletions; adult TK2d with late onset and mtDNA multiple deletions.^97^

Nucleos(t)ide supplementation therapy was demonstrated effective and safe in ameliorating the clinical, biochemical, and molecular genetics phenotype, but it does not represent a definitive cure since mice still died at 60 days with the highest doses.^34^^,^^98^ *hTK2*-AAV9 and *hTK2*-AAV2, under the control of CBA promoter and CMV enhancer, were administered in three different cohorts of *TK2* H126N mice (Table 1).^34^^,^^49^ The first one was treated with a retro-orbital injection of 4 × 10^10^ vg of AAV9 at PD1; the second one with a higher dosage of 4 × 10^11^ vg under the same conditions; a third group was administered with 2.1 × 10^11^ vg of AAV9 at PD1 and with 1.05 × 10^11^ of AAV2 at PD29, plus a subgroup was co-treated with 520 mg/kg/day each of deoxycytidine (dC) and deoxythymidine (dT) from PD21. The survival was prolonged up to a median of 39 days in the first group and 88.5 days in the second group. Levels of *TK2* mRNA in high-dose AAV9 were found in skeletal muscle, brain, and liver, and they remained stable until 18 months of age. However, the transduction efficiency was low in the kidneys. TK2 activity was rescued in AAV9-treated mice in muscle (40-fold higher than WT), brain (140%), and liver (93%), but not in the kidney at PD29. The activity began to decrease at PD60 in the brain and muscles, while it persisted unvarying in the liver. Mitochondrial DNA levels were comparable between AAV9-treated and WT mice. In addition, AAV9-treated mice displayed severe mtDNA depletion in the kidney, ultimately resulting in renal dysfunction. To mitigate potential immunogenicity, the authors improved the treatment strategy by administering a second injection of the AAV2 serotype. A combination of AAV9+AAV2 showed only partially ameliorated kidney dysfunctions; however, the lifespan was significantly prolonged to 120 days. However, the best results were obtained in the sub-cohort of mice treated with dC and dT, achieving a median age of 181 days, with a peak of 481 days. This sub-cohort also showed a greater weight gain and a higher mtDNA rescue in the liver, thereby emerging as the most effective strategy.^35^

## Translation into human-use: clinical trials

**Figure 2:**
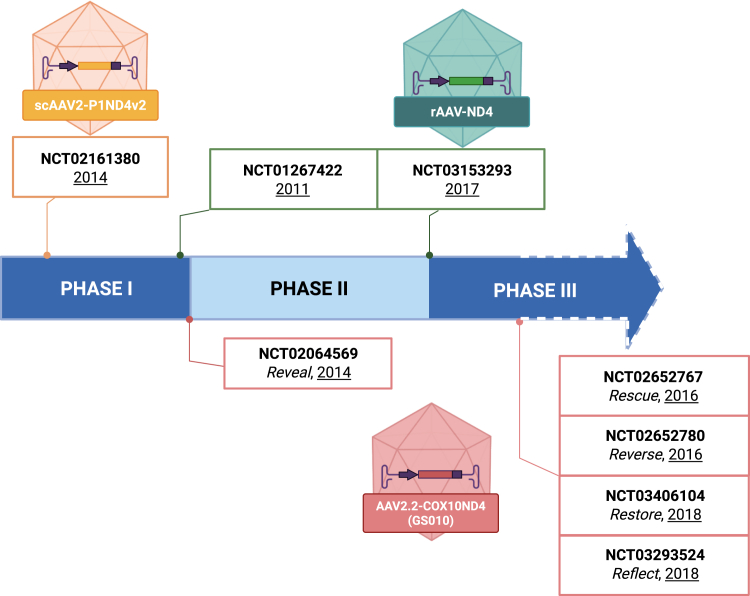
Timeline of clinical trials of current gene therapy for *ND4-associated* disease

Despite the high number of applications of gene therapies *in vivo* and *in vitro*, clinical trials have been carried out only for LHON with the m.11778G>A mutation in the *ND4* gene (Figure 2). An allotopic gene therapy approach with the mitochondrial sequence of the gene *ND4* translated into the nuclear genetic code was tested, and three different clinical trial programs were opened: in the USA, NIH sponsored the NCT02064569, in Europe, GenSight Biologics started the NCT0264569, and in China, NeurOphth financed NCT01267422.

In 2014, in the USA, a phase 1 open-label study (NCT02161380) experimented with scAAV2-P1ND4v2 viral vector in one eye of five patients. A follow-up of 90–180 days reported no safety issues.^50^ Then, the clinical trial ensued with unilateral injection in three different groups: six patients with a longer than 12 months bilateral loss of vision, six patients with a less than 12 months bilateral loss of vision, and two patients with a unilateral visual loss. In the 12 months of follow-up, improvements were appreciated in both eyes.^99^ Twenty-seven patients were monitored until 36 months, and visual acuity was improved with an incidence of uveitis without sequelae. The safety profile was considered good by the authors as the advent of uveitis was attributed only to the higher dose (1 × 10^10^ vg/eye) and not the patient group.^100^

The NCT01267422 clinical trial, carried out in China, was an open-label study started in December 2011. The primary endpoint was the recovery of best-corrected visual acuity (BCVA). As secondary endpoints, authors monitored changes in liver and kidney function, in the visual field, and the production of AAV2-antibody. Gene therapy was based on a rAAV-ND4 charged with *hND4*. Nine children and adults with confirmed m.11778G>A were recruited, and eight of them received a unilateral intravitreal injection. Doses were 5 × 10^9^ vg/0.05 mL for patients younger than 12 years of age (three patients) and 1 × 10^10^ vg/0.05 mL for the older group (six patients). One patient of the older group received a second dose 1 year later. After 36 months, four patients (three in the older group and one in the younger group) displayed an improved visual acuity in both eyes. In addition, the visual field was ameliorated in five patients (three of them belonged to the lower dose group and two to the higher one). In the 7 years of follow-up, eight patients still presented a significant improvement in BCVA with no adverse events (no data available for the long term in one patient). Phase 2 and 3 trials (NCT03153293) were initiated in 2017 by administering a single injection of 1 × 10^10^ vg in 0.05 mL, showing a rapid improvement in visual acuity.^101^^,^^102^ In 2018, a new clinical trial was conducted to evaluate patients whose symptoms onset within 3 months. This clinical trial is still ongoing (NCT03428178).

In 2014, Europe GenSight Biologics started the clinical phase 1–2 trial named REVEAL (NCT02064569), assessing the safety and tolerability of AAV.2.2-COX10ND4 (GS010). The study administered the worse-seeing eye with an intravitreal injection in 19 patients. REVEAL included four different doses of vector (9 × 10^9^, 3 × 10^10^, 9 × 10^10^, and 1.8 × 10^11^ vg/eye). The primary endpoint was the evaluation of the safety and tolerability of escalating doses. There were no serious adverse events in any of the doses, showing an increased BCVA.^103^ In 2016, two phase 3 clinical trials aimed to evaluate the efficacy of the vector in patients treated with a single intravitreal injection of 9 × 10^10^ vg in 90 μL in only one eye. Clinical trials were named RESCUE (NCT02652767) for the follow-up until 6 months and REVERSE (NCT02652780) for those from 6 to 12 months. A RESCUE (multicenter, randomized, double-masked, sham-controlled) clinical trial was carried out on 38 participants, and 24 weeks after the treatment, patients reported advancements in both of their eyes.^104^ REVERSE recruited 37 subjects older than 15 years, and at 96 weeks of follow-up, 68% of patients showed an improvement in BCVA in the treated eyes and 78% in both of their eyes.^105^

To further investigate the efficacy of double or single injection, in 2018, the phase III clinical trial REFLECT (NCT03293524) was initiated. A total of 98 patients received 9 × 10^10^ vg in 90 μL GS010, subgroups as follows: a single eye administration in 50 patients and both eyes administration in 48 patients. Results showed great improvements in the double injection group, although a not significant increase in visual acuity was also reported in the placebo eye.^106^

RESTORE (NCT03406104) recruited 61 subjects among the RESCUE and REVERSE to estimate the long-term follow-up. Patients were injected with 9 × 10^10^ vg in only one eye, and results after 52 months showed improved quality of life and visual acuity in both eyes.^107^

## Conclusions and future perspectives

Gene therapy targeted to specific gene defects or mRNA drugs modulating common pathways holds the promise to cure mitochondrial disorders. Our review highlights challenges in identifying the serotype and promoter able to deliver the gene therapy with the same efficiency and safety to different affected tissues; the need for double and multiple located injections for delivering the gene therapy; the limits in restoring the biochemical mitochondrial respiratory chain activity defects and sustaining the efficacy from young to adult age *in vivo* models; the potential limited translational value when comparing the gene therapy in small animals with different genetic background with large animals or humans. Organoids derived from patients’ biological samples and/or novel disease models reproducing with more fidelity the human diseases are needed for defining in detail the vector characteristics and gene therapy timing and doses for obtaining the highest efficacy and translational values with no or minimum side effects, including potential immunogenicity. Novel delivery methods, such as the use of nanoparticles with small molecules or RNA therapeutics as cargo, should be considered as future perspectives. Nanomedicines can protect the compound from degradation, reach different target tissues by modifying the surface characteristics, and cross the blood-brain barrier with efficiency. Nanomedicines have already been trialed in human diseases, and some of them have been recently translated into clinical trials for human use. Moreover, a new frontier of gene editing approaches with cytidine or adenosine deaminase able to correct a single nucleotide variant defect may open the way for more effective and safe treatment.

Despite the fact that some approaches presented in our review have demonstrated to be able to reduce those limits and challenges, very few of them have been manufactured as GMP products and translated into clinical trials for humans. Mitochondrial disorders face the same limits as other rare diseases in the development of gene therapy from pre-clinical and clinical studies to manufacturing and market authorization. The investment required to take advanced therapy medicinal products (ATMPs) to market authorization is estimated at 1 billion dollars. The complexity of the manufacturing, the stringent regulatory requirements, together with the cost and the low potential business return, make ATMP less attractive and/or not feasible for pharma companies.^108^ Although altogether mitochondrial disorders have an incidence of 1 out of 5,000 live births, when we consider gene therapy targeting a specific defective protein, the cohort of patients potentially treatable may include a few dozen cases. In addition to that, the clinical variability and the absence of unique biomarkers and/or functional tests for trial readiness still represent a limit for the design of clinical trials. Natural history studies, patients’ registries, and the advent of Al and digital technologies may pave the way for identifying novel outcome measurements for clinical trials, whereas manufacturing infrastructures and technological advances might, in the near future, reduce the cost of ATMP production bringing hope to translate more gene therapy approaches into clinical practice for mitochondrial diseases.

## Acknowledgments

C.G., S.S., and S.C. are supported by the European Union - NextGenerationEU, Mission *4 Component 2 – CUP:* B93D21010860004, Spoke n.2 “Genetics.” Figures were created by Biorender.

## Author contributions

Conceptualization, C.G.; writing – original draft, C.G., S.S., and S.C.; visualization, C.G., S.S., and S.C.; funding acquisition, C.G.

## Declaration of interests

C.G., S.S., and S.C. declare no competing interests.
